# Stomatal behaviour and water relations in ferns and lycophytes across habits and habitats

**DOI:** 10.1093/aobpla/plae041

**Published:** 2024-07-20

**Authors:** Kyra A Prats, Adam B Roddy, Craig R Brodersen

**Affiliations:** School of the Environment, Yale University, 195 Prospect St, New Haven, CT 06511, USA; New York Botanical Garden, 2900 Southern Blvd, Bronx, NY 10458, USA; Institute of Environment, Department of Biological Sciences, Florida International University, 11200 SW 8th Street, OE 148, Miami, FL 33199, USA; School of the Environment, Yale University, 195 Prospect St, New Haven, CT 06511, USA

**Keywords:** anatomy, ferns, lycophytes, mesic, Pteridaceae, stomata, water relations, xeric

## Abstract

Stomatal anatomy and behaviour are key to managing gas exchange fluxes, which require coordination with the plant vascular system to adequately supply leaves with water. Stomatal response times and regulation of water loss are generally understudied in ferns, especially across habits (i.e. epiphytic and terrestrial) and habitats (i.e. wet mesic and dry xeric environments). Our objectives were to (i) determine if hydraulic and anatomical traits that control water use are correlated with their habitats (i.e. xeric, mesic) and habits (i.e. epiphytic, terrestrial) for ferns and lycophytes across taxa, and (ii) explore how those traits and others like average leaf water residence time correlate with stomatal function using a subset of closely related species. Epiphytic species had lower vein densities than terrestrial species, while xeric species had higher vein densities than mesic species. Xeric ferns also had smaller stomata than mesic ferns but had similar stomatal densities. Further, in a subset of mesic and xeric ferns, the xeric ferns had higher maximum stomatal conductance and water content, as well as shorter average stomatal opening responses to light intensity, but stomatal closing times did not differ. Finally, shorter stomatal opening and closing responses were correlated with shorter water residence time. Our study highlights anatomical and physiological differences between ferns and lycophytes, which may partially explain habitat preference based on their optimization of light and water.

## Teaser

Ferns and lycophytes that occupy wet (mesic) and dry (xeric) habitats and grow as terrestrial or epiphytic plants had differing stomatal and vein anatomy, features that manage gas exchange fluxes and coordinate to control water supply. Within a subset of closely related ferns, the xeric species had faster stomatal opening response to light, but closing responses were similar. Our data show there are anatomical and physiological differences between these studied ferns and lycophytes across habits (epiphytic and terrestrial) and habitats (xeric and mesic), which at least in part likely contribute to environmental preference in their acquisition and utilization of light and water.

## Introduction

The flux of CO_2_ and H_2_O into and out of leaves is critical for sustaining photosynthesis and occurs primarily via stomata embedded in leaf surfaces. Stomata, which evolved around 400 mya (Edwards *et al.* 1998; Raven 2002), are formed by pairs of specialized cells that open and close in response to environmental conditions, such as light and humidity, to regulate gas exchange. Thus, environmental, physiological and anatomical factors influencing stomatal function directly impact not only the exchange of carbon and water at the scale of individual plants (Brodribb and McAdam 2011) but also the cycling of carbon and water both regionally and globally (Hetherington and Woodward 2003). While many studies have characterized stomatal function in trees and other flowering plants, the stomatal dynamics of ferns and lycophytes—diverse groups of plants with roughly 12 000 species (PPG1 *et al.* 2016)—have been less studied, despite ferns and lycophytes being some of the earliest plants with both a vascular system and stomata. Furthering our understanding of fern and lycophyte stomatal dynamics could provide insight into evolution of stomatal control across other plant lineages.

Stomata open and close in response to environmental stimuli such as changes in light intensity, CO_2_, relative humidity, VPD or soil water potential (Hetherington and Woodward 2003; Outlaw 2003; Franks and Farquhar 2007). Previous work has also shown that stomatal size influences guard cell dynamics, with smaller stomata opening and closing faster than large stomata in response to environmental stimuli (Hetherington and Woodward 2003; Franks and Farquhar 2007; Drake *et al.* 2013). One hypothesis for the faster-observed responses in smaller stomata is the high surface area to volume ratio compared to large guard cells (Franks *et al.* 2009; Drake *et al.* 2013). However, this may be true only within certain genera, as other studies have not found a relationship between stomatal size and opening or closing speed (Elliott-Kingston *et al.* 2016; McAusland *et al.* 2016). Available data show ferns and lycophytes often have large stomata occurring at low densities, especially compared to angiosperms (Franks and Beerling 2009; Carins Murphy *et al.* 2017), although there are exceptions for both ferns and angiosperms (e.g. some ferns have small stomata (Kübarsepp *et al.* 2020)).

Notably, stomatal closure in response to water deficit in most ferns and lycophytes is thought to occur independently of the signalling molecule abscisic acid (ABA) used by angiosperms, which is thought to make their stomatal responses to water deficit more predictable (Brodribb and McAdam 2011, 2017; McAdam and Brodribb 2012; Creese *et al.* 2014). Ferns have been shown to respond most quickly to VPD and to have slower responses to light and CO_2_ (Kübarsepp *et al.* 2020). Indeed, fern stomata typically do not close as effectively at low light levels compared to angiosperms, resulting in excessive water loss and low water use efficiency (McAdam and Brodribb 2012).

Water availability in leaves is coordinated with stomatal dynamics and transpiration. For leaves to meet evaporative demand and replace transpirational losses without stomatal closure, water must be supplied throughout leaves by veins and/or be available as stored water. Interspecific differences in vein density might allow for certain species to meet this evaporative demand better than others, where higher vein density minimizes the hydraulic pathway between veins and evaporative surfaces inside leaves. Additionally, average water residence time in leaves indicates the average amount of time water stays in the bulk leaf water pool and is a function of leaf water content divided by transpiration, capturing the time to desiccation within the leaf (Farquhar and Cernusak 2005; Roddy *et al.* 2016, 2023). Thus, leaf water residence times may align with how rapidly stomata respond to changes in atmospheric conditions (Simonin *et al.* 2013). For example, slower stomatal closure times could be compensated by higher capacitance and longer residence time that would prevent desiccation of the inner leaf. Relationships between leaf water residence time and stomatal dynamics are unexplored in ferns.

To address these knowledge gaps related to fern and lycophyte stomatal dynamics and water relations, we developed two specific goals: (i) to determine if key hydraulic and anatomical traits that regulate water use are correlated with ecological habitats (i.e. xeric, mesic) and habits (i.e. epiphytic, terrestrial), and (ii) to explore how those traits correlate with stomatal function for a subset of closely related ferns. For this last goal, we characterized stomatal responses to step changes in light intensity and VPD and also coupled these stomatal response measurements with pressure-volume (PV) curves and diurnal measurements of gas exchange and water residence times to elucidate how short-term stomatal responses may be related to *in situ* gas exchange dynamics. We hypothesized if there is a relationship between guard cell size and stomatal opening or closing times, as has been shown for angiosperms (Drake *et al.* 2013), then among the subset of closely related ferns, we would expect the species with smaller guard cells to have faster stomatal response times. Second, we expected to find patterns across growth habits and habitats. Given the persistent risk of dehydration and water limitation in dry environments, ferns occupying those habitats should have higher vein densities to supply leaves with water because of high evaporative demand. Lastly, for a subset of fern taxa we predicted fast stomatal response times would correlate with fast leaf water residence times.

## Materials and Methods

### Species selection for anatomy

We selected 30 fern and 8 lycophyte species from 12 fern and 2 lycophyte families to obtain broad coverage of extant fern and lycophyte diversity in our anatomical data (Table 1). These species span mesic (wet with ample moisture), xeric (dry or rocky with limited moisture) habitats, epiphytic (rooted to branches or trunks of trees), and terrestrial (rooted to the ground) habits. We consulted the literature when assigning a species’ xeric, mesic, terrestrial, and epiphytic categorization (Table 1). In Table 1, we also indicate which species were used for each anatomical or physiological measurement to help summarize our methods.

**Table 1.** All species of ferns and lycophytes we measured in experiments. There are two lycophyte families (Lycopodiaceae and Selaginellaceae) and 12 fern families (Aspleniaceae, Cibotiaceae, Cyatheaceae, Davalliaceae, Dryopteridaceae, Lygodiaceae, Nephrolepidaceae, Osmundaceae, Polypodiaceae, Psilotaceae, Pteridaceae, and Tectariaceae) represented. All species listed were used in stomatal anatomy and vein density measurements; the subset of species used for stomatal response times and pressure–volume curves are denoted with *, while **†** indicates the subset of species used for average water residence time.

Species	Clade	Family	Habitat	Origin	Source
*Actiniopteris semiflabellata*	Fern	Pteridaceae	Terrestrial lithophyte, xeric (Vaganov and Shmakov 2016)	Africa	NYBG Nolen Greenhouse
Adiantum capillus-veneris* ^†^	Fern	Pteridaceae	Terrestrial, mesic (Gleason and Cronquist 1963)	Cosmopolitan	YSE Greenhouse (sourced from Van Wilgen’s)
*Adiantum formosum*	Fern	Pteridaceae	Terrestrial, mesic (Cundall 2008)	Australia and New Zealand	NYBG Nolen Greenhouse
*Adiantum pulverulentum*	Fern	Pteridaceae	Terrestrial, mesic (Tuomisto *et al.* 1998)	Central and South America	NYBG Nolen Greenhouse
*Asplenium nidus*	Fern	Aspleniaceae	Epiphytic, tropical mesic (Zhang *et al.* 2010)	SE Asia, Australia, and Eastern Africa	NYBG Nolen Greenhouse
*Asplenium surrogatum*	Fern	Aspleniaceae	Epiphytic, subtropical mesic (Xu *et al.* 2020)	Australia	NYBG Nolen Greenhouse
Astrolepis sinuata* ^†^	Fern	Pteridaceae	Terrestrial, xeric (Rothfels *et al.* 2008)	Americas	NYBG Nolen Greenhouse and YSE Greenhouse
*Bolbitis heteroclita*	Fern	Dryopteridaceae	Terrestrial, tropical mesic (Xiang *et al.* 2011)	SE Asia and Oceania	NYBG Nolen Greenhouse
*Campyloneurum xalapense*	Fern	Polypodiaceae	Epiphytic, tropical mesic (Acebey *et al.* 2017)	Central America	NYBG Nolen Greenhouse
Cheilanthes distans* (nom. cons.)	Fern	Pteridaceae	Terrestrial lithophyte, xeric (Rothfels *et al.* 2008)	Australia and New Zealand	YSE Greenhouse (sourced from Plant Delights)
*Cheilanthes viridis* (nom. cons.)	Fern	Pteridaceae	Terrestrial lithophyte, xeric (Rothfels *et al.* 2008)	Africa	YSE Greenhouse (sourced from Plant Delights)
*Cibotium glaucum*	Fern	Cibotiaceae	Tree fern, mesic (Becker 1984)	Hawai’i	NYBG Nolen Greenhouse
Coniogramme japonica* ^†^	Fern	Pteridaceae	Terrestrial, evergreen, mesic (Wang *et al.* 2019)	East Asia	YSE Greenhouse (sourced from Plant Delights)
*Cyrtomium falcatum*	Fern	Dryopteridaceae	Terrestrial, mesic (Chung *et al.* 2012)	East Asia	NYBG Nolen Greenhouse and YSE Greenhouse
*Davallia heterophylla*	Fern	Davalliaceae	Epiphytic, tropical mesic (Magtoto and Austria 2017)	SE Asia and Oceania	NYBG Nolen Greenhouse
*Drynaria speciose*	Fern	Polypodiaceae	Epiphytic, tropical mesic (Boonkerd 2006)	SE Asia and Australia	NYBG Nolen Greenhouse
*Drynaria splendens*	Fern	Polypodiaceae	Epiphytic, tropical mesic (Coritico *et al.* 2020)	SE Asia and Australia	NYBG Nolen Greenhouse
*Huperzia phlegmaria*	Lycophyte	Lycopodiaceae	Epiphytic, tropical mesic (Rusea *et al.* 2009)	SE Asia and Oceania	NYBG Nolen Greenhouse
*Lygodium circinnatum*	Fern	Lygodiaceae	Climbing fern (terrestrial), mesic (Lott *et al.* 2003)	SE Asia and Oceania	NYBG Nolen Greenhouse
*Microsorum musifolium*	Fern	Polypodiaceae	Epiphytic, tropical mesic (Testo and Sundue 2014)	Oceania	NYBG Nolen Greenhouse
*Myriopteris cucullans*	Fern	Pteridaceae	Terrestrial, xeric (Grusz and Windham 2013)	Central America	NYBG Nolen Greenhouse
*Myriopteris lanosa*	Fern	Pteridaceae	Terrestrial lithophyte, xeric (Grusz and Windham 2013)	Eastern North America	YSE Greenhouse (sourced from Plant Delights)
*Nephrolepis brownii*	Fern	Nephrolepidaceae	Terrestrial, mesic (Magtoto and Austria 2017)	Asia	NYBG Nolen Greenhouse
*Osmunda japonica*	Fern	Osmundaceae	Terrestrial, mesic (Tsutsumi *et al.* 2015)	Asia	YSE Greenhouse (sourced from Plant Delights)
*Pellaea falcata*	Fern	Pteridaceae	Terrestrial, mesic (Kirkpatrick 2007)	Australia	NYBG Nolen Greenhouse
*Phlegmariurus nummularifolius*	Lycophyte	Lycopodiaceae	Epiphytic, tropical mesic (Field and Bostock 2013)	Oceania	NYBG Nolen Greenhouse
*Pleopeltis lepidopteris*	Fern	Polypodiaceae	Terrestrial, mesic (Filipin *et al.* 2017)	Brazil	YSE Greenhouse (sourced from Plant Delights)
Polystichum acrostichoides* ^†^	Fern	Dryopteridaceae	Terrestrial, mesic wintergreen (Soltis *et al.* 1990; Prats and Brodersen 2020)	North America	YSE Greenhouse (sourced from Yale Myers Forest)
*Psilotum complanatum*	Fern	Psilotaceae	Epiphytic, tropical mesic (Coritico and Amoroso 2020)	Oceania, Hawai’i	NYBG Nolen Greenhouse
*Psilotum nudum*	Fern	Psilotaceae	Epiphytic or lithophyte, mesic (Imdorf *et al.* 1994)	Tropics (Americas, Africa, Asia, Australia)	NYBG Nolen Greenhouse
*Pyrrosia Heteractis*	Fern	Polypodiaceae	Epiphytic or lithophyte, tropical mesic (Kotrnon *et al.* 2007)	Asia	NYBG Nolen Greenhouse
*Selaginella braunii*	Lycophyte	Selaginellaceae	Terrestrial, xeric (Zhou *et al.* 2016)	Asia, North America	NYBG Nolen Greenhouse
*Selaginella kraussiana*	Lycophyte	Selaginellaceae	Terrestrial, mesic (Brownsey and Perrie 2018)	Africa, New Zealand	NYBG Nolen Greenhouse
*Selaginella moellendorffii*	Lycophyte	Selaginellaceae	Terrestrial, mesic (Brownsey and Perrie 2018)	Asia, New Zealand	YSE Greenhouse (sourced from Plant Delights)
*Selaginella serpens*	Lycophyte	Selaginellaceae	Terrestrial, mesic, tropical (Tryon and Tryon 1982)	Caribbean	NYBG Nolen Greenhouse
*Selaginella uncinata*	Lycophyte	Selaginellaceae	Terrestrial, mesic (Jagels 1970)	Asia	YSE Greenhouse (sourced from Plant Delights)
*Sphaeropteris* *Cooperi*	Fern	Cyatheaceae	Tree fern, mesic (Wood 2008)	Australia	NYBG Nolen Greenhouse
*Tectaria gemmifera*	Fern	Tectariaceae	Terrestrial, mesic (Lovett and Thomas 1986)	Africa	NYBG Nolen Greenhouse
*Zealandia pustulata*	Fern	Polypodiaceae	Epiphytic, subtropical mesic (Brownsey and Perrie 2014)	Australia and New Zealand	NYBG Nolen Greenhouse

^*^Species used for stomatal response times and pressure–volume curves.

^†^Species used for average water residence.

Some of these species were grown in 16 cm wide × 18 cm tall pots in a greenhouse at the Yale School of the Environment (YSE) (see Table 1 for details on plant source) where they were watered a total of 0.6 L (mesic species) or 0.3 L (xeric species) daily based on inherent differences in the watering needs. Plants were fertilized with Osmocote (ICL Specialty Fertilizers, Summerville, SC, USA) every 3 months. Conditions in the YSE Greenhouse were kept at approximately 24 °C and 45% relative humidity, with PPFD reaching 40–50 µmol m^−2^ s^−1^ (LI-250A, Li-Cor, Lincoln, NE, USA). The remaining species were sampled only for anatomical traits at the New York Botanical Garden (NYBG) Nolen Greenhouse. We constructed a cladogram depicting relationships between our sampled species (see Supporting Information—Fig. S1). To do so, we consulted established fern and lycophyte phylogenies (Schuettpelz and Pryer 2007; Schuettpelz *et al.* 2007; PPG1 *et al.* 2016) and used the R packages ‘ape’ (Paradis and Schliep 2019), ‘phangorn’ (Schliep *et al.* 2017), ‘phytools’ (Revell 2012), and ‘geiger’ (Pennell *et al.* 2014). We also used the fern tree of life (FTOL) phylogeny (Nitta *et al.* 2022) for phylogenetic analyses involving only the ferns (see statistics section of the methods).

### Species selection for physiology subset

We measured a suite of physiological traits on at least three individuals from a subset of five species growing in the YSE greenhouse, including two xeric-adapted (*Astrolepis sinuata* Sw. and *Cheilanthes distans* Colenso from the Cheilanthoideae) and two mesic-adapted (*Coniogramme japonica* Ogata from the Cryptogrammoideae and *Adiantum capillus-veneris* from the Adiantoideae/Vittarioideae) ferns from the Pteridaceae, and one mesic-adapted species from the Dryopteridaceae (*Polystichum acrostichoides*) (Table 1). For these five species we measured rates of stomatal response to step changes in light intensity and VPD, PV curves, and diurnal changes in stomatal conductance and leaf water content. *C. distans* was not included in diurnal measurements of gas exchange and water content because the pinnae of this species were too dissected to be sealed inside the LI-600 porometer. We preferred to use the LI-600 rather than the LI-6800 for these measurements due to the number of samples and time points throughout the day; the efficiency of the LI-600 allowed us to keep our measurement time points consistent across samples.

### Vein and stomatal anatomy

Anatomical traits were measured on individuals cultivated in both at the NYBG Nolen Greenhouse and the YSE Greenhouse. To measure stomatal anatomy and vein density, at least two pinna sections from the middle lamina (~1 cm^2^, including veins) from each plant were cleared in 5% w/v sodium hydroxide for at least two weeks (Gardner 1975). Samples that had not cleared completely were further cleared in a 3% v/v bleach solution (Clorox, Oakland, CA, USA) for a few minutes to hours (depending on the sample), then rinsed in distilled water overnight. Samples were stained in a ~3:10 mixture of 1% w/v toluidine blue (RICCA Chemical Company, Arlington, TX, USA) in water for 15 min before being rinsed in distilled water and placed in 70% v/v ethanol overnight. Next, samples were extracted from ethanol using tweezers, mounted on microscope slides with Cytoseal™ 60 Mounting Solution (Thermo Fisher Scientific, Waltham, MA, USA), and imaged at 4×, 20× (for density measurements), and 40× or 50× (for stomatal anatomy) magnification objectives on a compound microscope (Olympus BX60 or Olympus BX51, Olympus America, Center Valley, PA, USA) outfitted with a Canon 6D digital SLR camera (Canon, Melville, NY, USA) or a 12.3 megapixel camera (High Quality Camera, Raspberry Pi Foundation, Cambridge, England, UK), respectively.

Vein and stomatal anatomy from sample images were measured in ImageJ (v2.0, U.S. National Institutes of Health, Bethesda, MD, USA). Stomatal density was calculated as number of stomata per mm^2^ within a certain area, and vein density was calculated as total length of veins per area measured (mm^2^). For *Selaginella* species, overlapping dorsal and ventral microphylls were both included in sectioning for vein density (Liu *et al.* 2022). Two species of *Psilotum* were excluded from statistical analyses of vein density given than measurements were of the stem and not a true leaf. Stomatal anatomy measurements were made following previous protocols for measuring stomatal pore length, width, and guard cell length (Sack and Buckley 2016). Stomatal size was calculated as guard cell length multiplied by guard cell width.

### Light response curves

Light response curves (*n* = 3 per species) were measured using a portable gas exchange system (LI-6800, Li-Cor, Lincoln, NE, USA) at photosynthetic photon flux densities (PPFDs) of 0, 5, 10, 20, 50, 75, 100, 250, 500, and 1000 µmol m^−2^ s^−1^. The LI-6800 was set to maintain the sample chamber at a [CO_2_] of 415 µmol mol^−1^, a VPD of 1.2 kPa, and a flow rate of 600 µmol s^−1^ while leaf temperature was controlled at 25 °C. Light response curves were fit using models (Marshall and Biscoe 1980) incorporated into the ‘photosynthesis’ package (Stinziano *et al.* 2021). We used light response curves to determine the light compensation point and the light intensity at saturated photosynthesis (see Supporting Information—Table S1). Those light saturation intensity values were considered when setting the light intensity step changes for stomatal responses. Individual light response curves are plotted in the Supporting Information (Fig. S2).

### Stomatal responses

We measured stomatal conductance in response to step changes in light intensity (both increases and decreases) on at least three individuals of *A. capillus-veneris*, *A. sinuata*, *C. japonica*, and *C. distans* using a portable gas exchange system (#LI-6800, Li-Cor, Lincoln, NE, USA). During these measurements, data were logged every 60 s. We followed previously published protocols for measuring stomatal responses to step changes in light intensity (Vialet-Chabrand *et al.* 2017; Eyland *et al.* 2021). We used a light intensity of 25 µmol m^−2^ s^−1^ photosynthetic photo flux density (PPFD) as a low light condition and 500 µmol m^−2^ s^−1^ PPFD for the higher light intensity for all species besides *A. capillus-veneris*, for which a high light of 250 µmol m^−2^ s^−1^ was used based on its light saturation point. The LI-6800 was set to maintain the sample chamber at a [CO_2_] of 415 µmol mol^−1^, a VPD of 1.2 kPa, and a flow rate of 600 µmol s^−1^. Leaf temperature was controlled at 25 °C. To begin light step changes, light intensity was set to 25 µmol m^−2^ s^−1^ PPFD for 10 min to achieve a steady state stomatal conductance. Next, light intensity was increased to 250 µmol m^−2^ s^−1^ for *A. capillus-veneris* and 500 µmol m^−2^ s^−1^ for all other species for 60 min to measure stomatal opening rates. Finally, light intensity was lowered back to 25 µmol m^−2^ s^−1^ for 30 min to measure stomatal closing rates. Since fern pinnae do not always fill the area of the chamber, pinnae were photographed after gas exchange measurements and area was measured in ImageJ (v2.0, U.S. National Institutes of Health, Bethesda, MD, USA) in order to recalculate gas exchange parameters. Rates of stomatal opening and closing to light were calculated by first using range normalization to normalize *g*_s_ to the maximum value for each species, and then calculating the slope of stomatal response from the initial step change (excluding any Li-6800 flow adjustments) to the point at which the *g*_s_ levelled off and the rate of change between measurements was ≤2%. We also measured stomatal responses to step changes in VPD; those methods and results can be found in the Supplement.

### PV curves

Pressure-volume (PV) curves were conducted on *A. capillus-veneris*, *A. sinuata*, *Coniogramme japonica*, *C. distans*, and *P. acrostichoides* for four leaves per species using a Scholander pressure chamber (Model 600, PMS Instruments, Albany, OR, USA). We followed established protocols for bench drying methods (Tyree and Hammel 1972; Koide *et al.* 2000; Sack *et al.* 2003; Sack and Pasquet-Kok 2011). Fronds were cut at the base of the stipe and rehydrated for half an hour. When needed, stipes were reinforced with parafilm to prevent fragile stipes from breaking. Stipe bases were cut again after initial rehydration and leaf material was placed inside Whirl-Pak bags with the stipe protruding; fronds remained like this for the duration of PV curve measurements (Whirl-Pak, Madison, WI, USA). As samples slowly desiccated over time inside the Whirl-Pak bags, sequential measurements of water potential and mass were made on each frond with a precision balance (Practum 224-1s, Sartorius, Gottingen, Germany). This process was repeated until fronds reached water potentials between −2.0 and −3.0 MPa and were visually wilted. Fronds were oven dried at 80 °C for 3 days to record dry mass. Water potential at turgor loss point (TLP), saturated water content (SWC), relative water content (RWC) at TLP, and absolute capacitance were determined for each sample using the ‘photosynthesis’ package in R (Stinziano *et al.* 2021).

### Average water residence time

Average leaf water residence time was measured on *A. capillus-veneris*, *A. sinuata*, *C. japonica*, and *Polystichum acrostichoides* on two separate days in May and June 2021 following previously established protocols (Roddy *et al.* 2018). Measurements were taken at five diurnal time points (7:30, 9:30, 11:30, 13:30, and 15:30) throughout both sampling days. At each time point, stomatal conductance and transpiration were measured using a portable leaf porometer (LI-600, Li-Cor, Lincoln, NE, USA). Entire pinnae were excised at the petiole base, sealed in humidified plastic bags, and weighed for fresh mass within ~20 min of sampling (Practum 224-1s, Sartorius, Gottingen, Germany). Pinnae were scanned for leaf area, oven dried at 80 °C for 3 days, then weighed once more for dry weight. Average water residence time—or the average time water stays in the bulk leaf pool—at each time point was calculated as:


$$\begin{array}{l} \tau_{wt}=\;\frac{W}{E} \end{array}$$ (1)

where *W* is pinna water content per leaf area (mol m^−2^) calculated from the difference between fresh mass and dry mass, *E* is transpiration rate (mol m^−2^ s^−1^) from porometer measurements, and *τ*_*wt*_ is leaf water residence time of water cycling through the leaf (seconds) as a function of *W* and *E* (Farquhar and Cernusak 2005; Roddy *et al.* 2018). Water residence times were converted to hours for easier interpretation.

### Statistics

All statistical analyses were conducted in R version 4.1.1, Foundation for Statistical Computing, Vienna). Linear regressions were run when comparing relationships between species means of anatomical traits (e.g., stomatal density and vein density); welch two-sample *t*-tests were conducted when comparing habitats (xeric and mesic species), and habits (epiphytic and terrestrial species) of both fern and lycophyte anatomical data. FTOL (Nitta *et al.* 2022) was also used to examine only the fern anatomical data within a phylogenetic context; using the package geomorph (Adams *et al.* 2024) and the function procD.pgls we performed phylogenetic ANOVAs based on 1000 permutations to examine differences between habitat and habit. Similarly, phylogenetic analyses were conducted on species means from the subset of five species when analysing differences between xeric and mesic stomatal response and PV curve traits. Finally, nonlinear least square regressions were conducted on the relationships between stomatal responses and leaf water residence time using ‘nls()’ with Delta 95% confidence intervals using the ‘nlraa’ package (Miguez *et al.* 2020).

## Results

### Leaf anatomy traits

**Figure 1.**
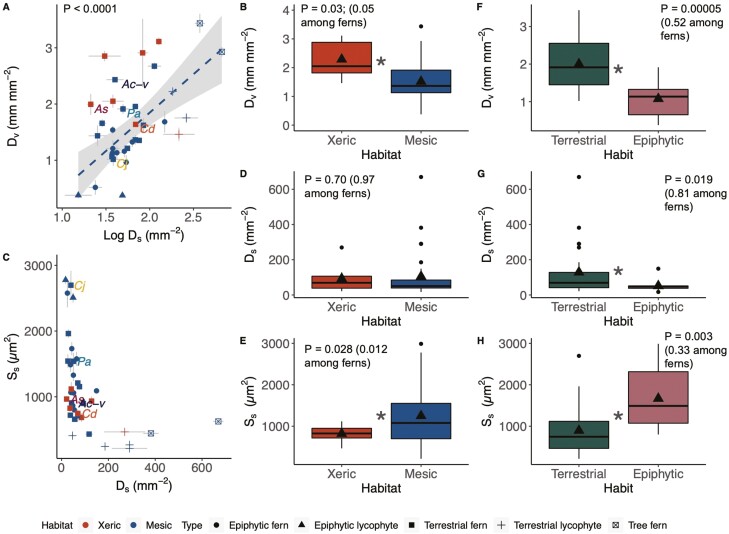
Relationships between fern and lycophyte (*n* = 38 species) stomatal and vein anatomy across species means (with shapes corresponding to habitat and type). (A) Log-transformed stomatal density (*D*_s_) was significantly correlated with vein density (*D*_v_) only in mesic ferns and lycophytes (dashed regression line only for mesic species). Labels are shown for the five species from stomatal response and water relations measurements (As = *Astrolepis sinuata*, Cd = *Cheilanthes distans*, Cj = *Coniogramme japonica*, Ac-v = *Adiantum capillus-veneris*, and Pa = *Polystichum acrostichoides*). (B) Xeric (*n* = 7 species) species had higher *D*_v_ than mesic (*n* = 31) species (significance denoted with *). (C) Fern and lycophyte *D*_s_ plotted against stomatal size (*S*_s_) shows small stomata can occur at a higher density, but large stomata occurred only at low densities; labels are again shown for the five species from stomatal response and water relationship measurements. (D) *D*_s_ was not significantly different between xeric and mesic ferns. (E) Xeric ferns had smaller stomata than mesic ferns (significance marked with *). Significant differences were found between terrestrial (*n* = 25 species) and epiphytic (*n* = 13 species) ferns and lycophytes with regard to vein density (F), stomatal density (G), and stomatal size (H). In the boxplots (B–H) the triangles represent the mean, the centre lines are the median, the box limits are the upper and lower quartiles, the whiskers are 1.5× interquartile range, and the points are potential outliers. *P*-values in B–H include both the fern and lycophyte data, and *P*-values in parentheses are from phylogenetic ANOVAs that only include the fern data. It is important to note that there are more mesic species than xeric species—as well as more terrestrial than epiphytic species—in this dataset.

Among ferns and lycophytes there was a 39-fold range in stomatal densities, from 17.1 (epiphytic lycophyte *Phlegmariurus nummularifolius* Blume (Ching)) to 669.6 mm^−2^ (tree fern *Cibotium glaucum* J. Sm.), with a mean of 102 mm^-2^ (± 127 SD).There was a 9-fold variation in fern and lycophyte vein density, from 0.37 mm^−1^ (epiphytic lycophyte *Huperzia phlegmaria* L.) to 3.44 mm^−1^ (tree fern *Sphaeropteris cooperi*). Mean fern and lycophyte vein density was 1.67 mm mm^−2^ (± 0.77 SD). A higher stomatal density among mesic ferns and lycophytes was correlated with a higher vein density (*P* < 0.0001; Fig. 1A and inset; see Supporting Information—Table S2); however, there was no relationship between stomatal density and vein density among xeric ferns (*P* = 0.85; see Supporting Information—Table S2). Xeric ferns had higher vein densities (2.29 ± 0.66 mm mm^−2^) than mesic (1.57 ± 0.73 mm mm^−2^) ferns and lycophytes (*P* = 0.03, Fig. 1B; Table 2), and epiphytes had lower vein densities than terrestrial species (*P* < 0.0001; Fig. 1F, Table 3). When lycophytes were excluded and only ferns were analysed with a phylogenetic ANOVA, xeric ferns still had higher vein densities (*P* = 0.05; see Supporting Information—Table S3) while epiphytes and terrestrial ferns were not significantly different (*P* = 0.52; see Supporting Information—Table S3).

**Table 2.** Means (± SD) of measured traits by xeric or mesic habitat and their significance based on Welch two-sample *t*-tests. Data are from the larger anatomy dataset that includes both ferns and lycophytes (*n* = 38).

Trait	Xeric	Mesic	*T*	DF	*P*-value
Stomatal density (mm^-2^)	92.89 ± 85.73	104.44 ± 136.31	-0.39	14.85	0.70
Stomatal size (µm^2^)	879.32 ± 158.29	1225.97 ± 782.89	−2.30	33.55	0.028 *
Stomatal pore length (µm)	22.42 ± 4.44	19.82 ± 7.79	1.15	11.60	0.27
Guard cell length (µm)	41.32 ± 5.85	42.62 ± 15.12	−0.37	19.93	0.72
Guard cell width (µm)	21.49 ± 4.05	26.19 ± 9.69	−1.99	17.81	0.06•
Vein density (mm mm^-2^)	2.29 ± 0.66	1.57 ± 0.73	2.51	10.05	0.03 *

**Table 3.** Means (± SD) of measured traits by epiphytic or terrestrial growth habit, and their significance based on Welch two-sample *t*-tests. Data are from the larger anatomy dataset (*n* = 38).

Trait	Epiphytic	Terrestrial	*T*	DF	*P*-value
Stomatal density (mm^-2^)	51.27 ± 31.90	128.85 ± 149.81	−2.48	27.94	0.019*
Stomatal size (µm^2^)	1668.00 ± 741.18	895.24 ± 571.13	3.38	21.76	0.003**
Stomatal pore length (µm)	23.47 ± 6.93	18.40 ± 7.13	2.17	27.71	0.039*
Guard cell length (µm)	52.05 ± 12.55	37.03 ± 11.95	3.65	25.93	0.001**
Guard cell width (µm)	31.18 ± 9.47	22.27 ± 7.41	3.04	22.04	0.006**
Vein density (mm mm^-2^)	1.07 ± 0.49	2.00 ± 0.70	−4.66	32.28	0.00005***

We found a 3.5-fold difference in stomatal length from 19.64 µm (± 1.34 SD, terrestrial lycophyte *Selaginella serpens*) to 69.53 µm (± 9.88 SD, terrestrial fern *C. japonica*). Small stomata can occur at a higher density, but large stomata occurred only at low densities (Fig. 1C). While there was no difference in stomatal density between xeric (92.89 ± 85.73 SD) and mesic ferns (104.44 ± 136.31 SD) (*P* = 0.70, Fig. 1D; Table 2), epiphytes had significantly lower stomatal densities than terrestrial species (*P* = 0.019; Fig. 1G, Table 3). Within a phylogenetic context, stomatal density between fern habitats and habits was similar (insignificant, see Supporting Information—Table S3).

Xeric ferns had significantly smaller stomata than mesic ferns (*P* = 0.028; Fig.1E; Table 2), and terrestrial ferns had significantly smaller stomata than epiphytic ferns (*P* = 0.003; Fig. 1H, Table 3). Xeric ferns analysed within a phylogenetic context still had statistically significantly smaller stomata than mesic ferns (*P* = 0.012; see Supporting Information—Table S3), but there were no differences between the sizes of terrestrial and epiphytic fern stomata (*P* = 0.33; see Supporting Information—Table S3). Similarly, guard cell length, width, and pore length did not vary between xeric and mesic species (Table 2). Stomatal guard cell length and width were strongly correlated (*P* < 0.00001; see Supporting Information—Fig. S3). All anatomical data from our dataset can be found in our Supplemental Spreadsheet.

### Stomatal responses

**Figure 2.**
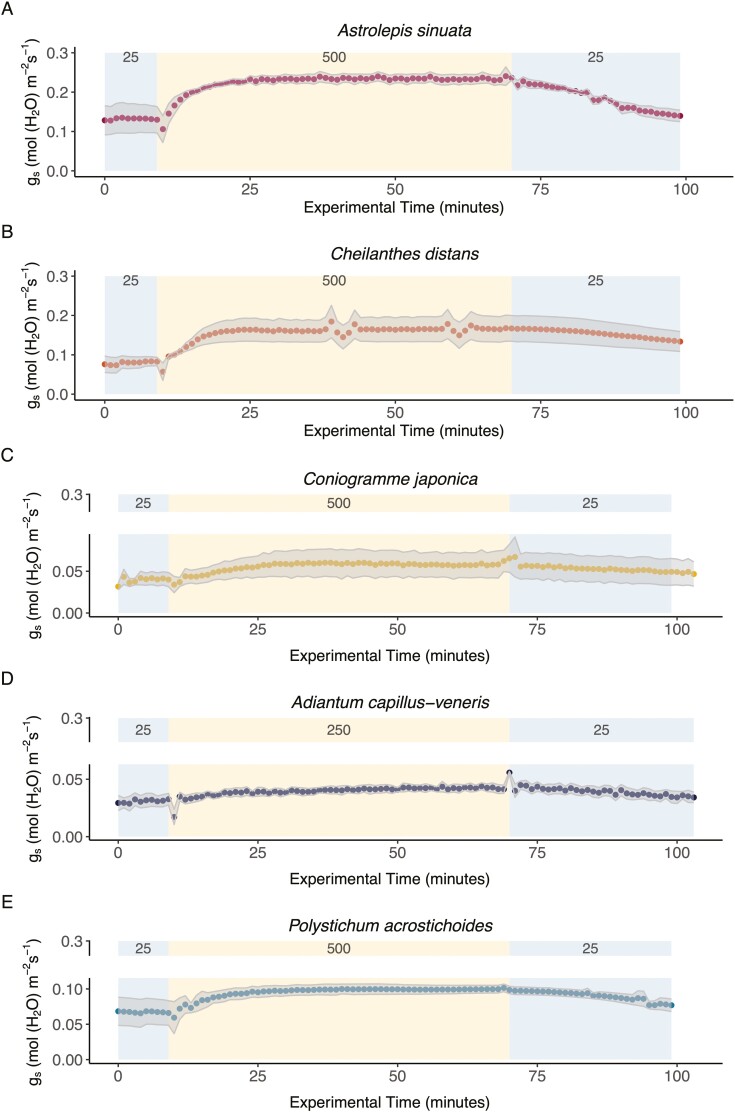
Average stomatal responses to step changes in light (A–E) across five species (xeric *Astrolepis sinuata* (*n* = 4), xeric *Cheilanthes distans* (*n* = 3), mesic *Coniogramme japonica* (*n* = 3), mesic *Adiantum capillus-veneris* (*n* = 3), and mesic *Polystichum acrostichoides* (*n* = 3)). Grey shading represents standard error around the mean (colored points). Background colors show the low and high light conditions, with corresponding numbers displaying the PPFD values. Axis breaks (Xu *et al.* 2021) are shown to provide more detail for the mesic species that had lower fluxes compared to the xeric species.

Stomatal responses to step changes in light intensity revealed subtle differences across the four Pteridaceae species and the one Dryopteridaceae species (Fig. 2A–E). Xeric species—*A. sinuata* and *C. distans*—had higher stomatal conductance than mesic *C. japonica*, *A. capillus-veneris,* and *P. acrostichoides*. Similar trends were found for stomatal responses to step changes in VPD (see Supporting Information—Figs. S4 and ).

**Figure 3.**
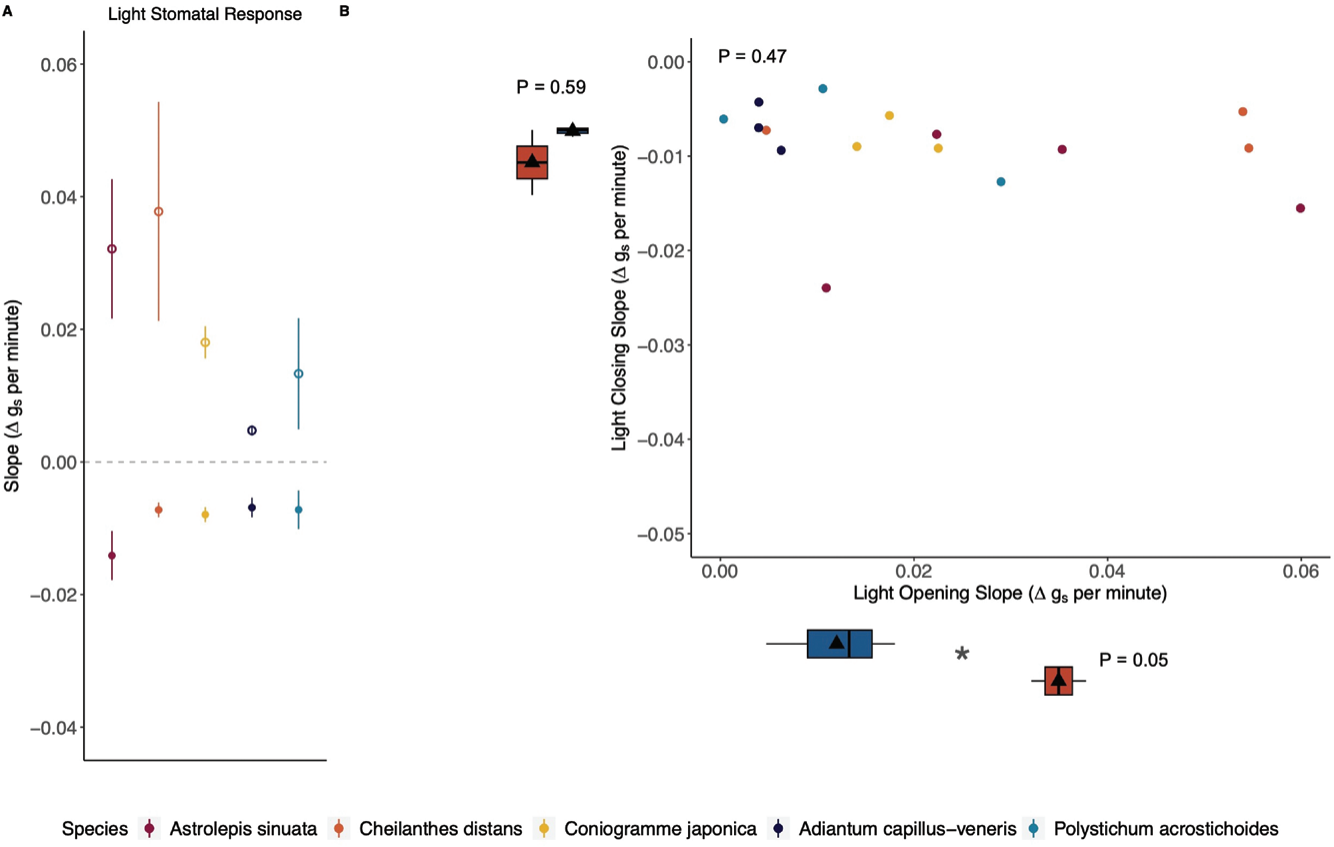
Stomatal response for the five species (xeric *Astrolepis sinuata* (*n* = 4), xeric *Cheilanthes distans* (*n* = 3), mesic *Coniogramme japonica* (*n* = 3), mesic *Adiantum capillus-veneris* (*n* = 3), and mesic *Polystichum acrostichoides* (*n* = 3)—displayed in this order in A from left to right). (A) Stomatal opening (open circles) and closing (closed circles) response to light, represented by the slope of the response (change in *g*_s_ per change in minute). Positive slopes signify stomatal opening, and negative slopes signify stomatal closing; the higher the absolute value, the larger the slope, and thus, the quicker the stomatal response. (B) Opening and closing rates in response to light was not correlated, but xeric (orange) species opened stomata significantly faster than mesic (blue) species (boxplot along *x*-axis, *P*-value from phylogenetic ANOVA); mesic and xeric stomatal closing slopes to light were not significantly different (boxplot along *y*-axis, *P*-value from phylogenetic ANOVA). In all xeric (*n* = 7) and mesic (*n* = 9) boxplots, the triangles represent the mean, the centre lines are the median, the box limits are the upper and lower quartiles, the whiskers are 1.5× interquartile range.

All species in our study opened stomata in response to increasing light intensity and closed stomata in response to decreasing light intensity, although the magnitude of the response varied by species (Fig. 2). While there was no relationship between stomatal opening and closing rates in response to light (*P* = 0.47, Fig. 3B), we found that xeric fern stomata opened faster in response to light compared to mesic species (phylogenetic ANOVA *P* = 0.05; Fig. 3B*x*-axis boxplot; see Supporting Information—Table S4). We found no significant differences between xeric and mesic species in their light-induced stomatal closure rate (*P* = 0.59; Fig. 3B*y*-axis boxplot, see Supporting Information—Table S4), although xeric *A. sinuata* had the fastest stomatal closing time. Light-induced stomatal opening or closing time was not correlated with stomatal pore length or width (see Supporting Information—Table S5).

### PV curves

**Figure 4.**
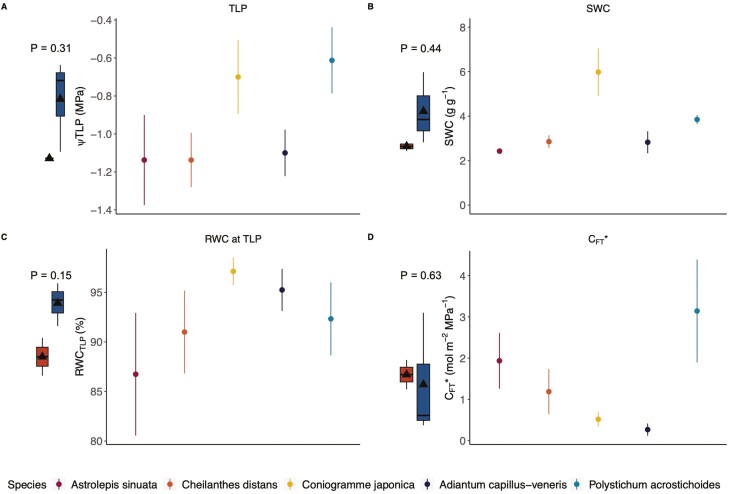
Water relations from PV curves for the five species (xeric *Astrolepis sinuata* (*n* = 4), xeric *Cheilanthes distans* (*n* = 4), mesic *Coniogramme japonica* (*n* = 4), mesic *Adiantum capillus-veneris* (*n* = 4), and mesic *Polystichum acrostichoides* (*n* = 4)—displayed in this order left to right). None of the PV curve traits (A) water potential at turgor loss point (TLP); (B) saturated water content (SWC); (C) relative water content at TLP (RWC_TLP_); and (D) absolute capacitance (*C*_FT_*)) differed significantly across species. Boxplots and *P*-values show the corresponding phylogenetic ANOVAs between the means of xeric and mesic species for each trait. In all xeric and mesic boxplots, the triangles represent the mean, the centre lines are the median, the box limits are the upper and lower quartiles, the whiskers are 1.5× interquartile range.

When analysed in a phylogenetic context, none of the traits derived from PV curves were significantly different between the xeric and mesic species (Turgor loss point (TLP) *P* = 0.31, Fig. 4A; saturated water content (SWC) *P* = 0.44, Fig. 4B; Relative water content at turgor loss point (RWC_TLP_) *P* = 0.15, Fig. 4C; Absolute capacitance (*C*_FT_*) *P* = 0.63, Fig. 4D; all statistics displayed in see Supporting Information—Table S4). Full plots of PV curves by species ( see Supporting Information—Fig. S6) and by individuals ( see Supporting Information—Fig. S7), as well as water potentials across drying time (see Supporting Information—Fig. S8) can be found in the Supplement.

### Diurnal stomatal conductance, water residence time, water content, and transpiration

**Figure 5.**
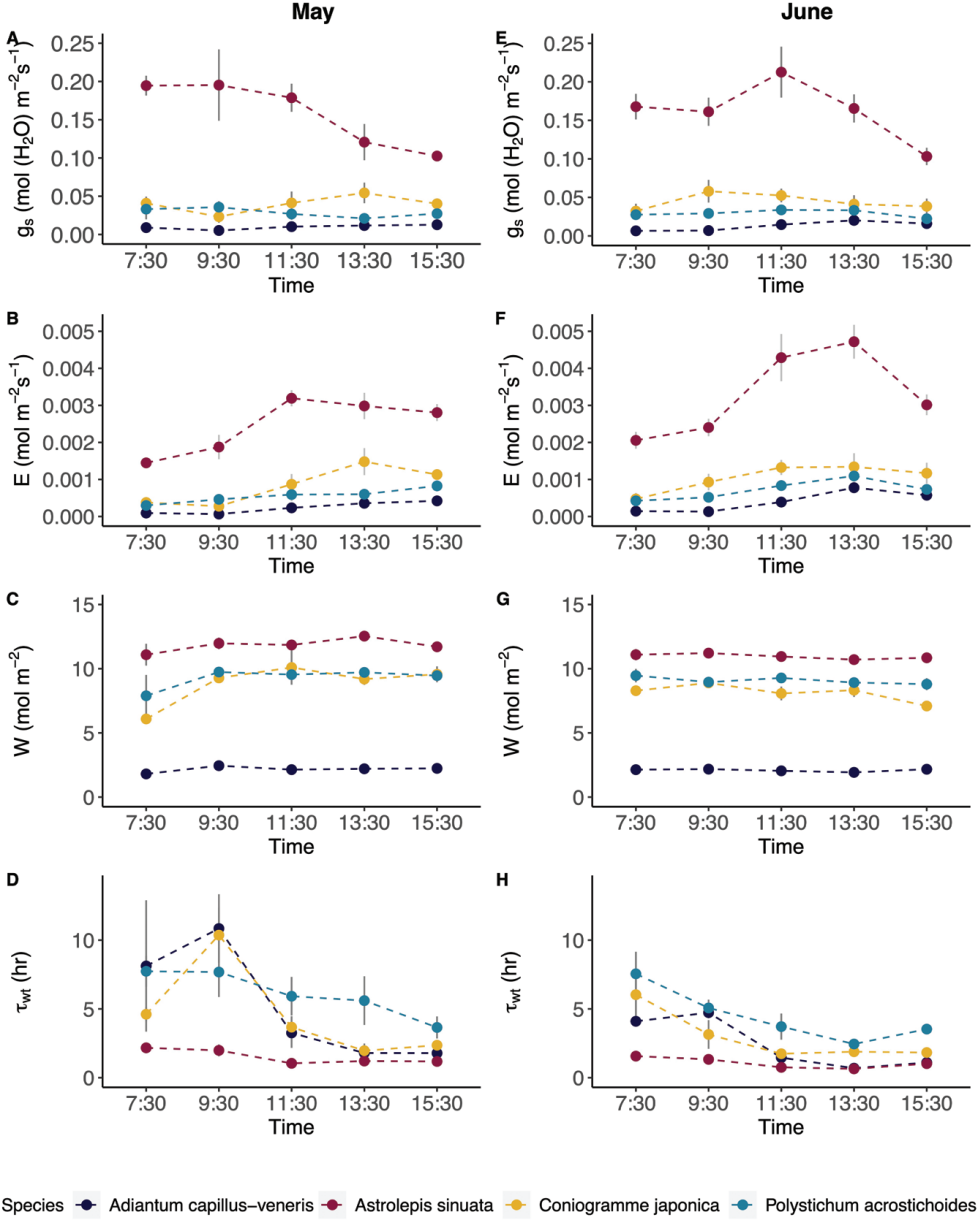
Mean stomatal conductance, transpiration (E), water content per area (W), and average water residence time (*τ*_wt_) across four species in May (A–D) and June (E–H) (xeric *Astrolepis sinuata* (*n* = 4), mesic *Coniogramme japonica* (*n* = 3), mesic *Adiantum capillus-veneris* (*n* = 3), and mesic *Polystichum acrostichoides* (*n* = 3)). Grey bars show the standard error around the means.

Xeric *A. sinuata* had the highest stomatal conductance (*g*_s_) of all species from diurnal measurements in both May and June (Fig. 5A and E, respectively). The other fern species had low *g*_s_ that did not fluctuate throughout the day. *A. sinuata* had the highest transpiration rate in May and June (Fig. 5B and 5F, respectively). Conversely, the three other fern species experienced more modest changes in already low diurnal transpiration rates. *A. sinuata* also had the highest water content for both months (Fig. 5C, May; Fig. 5G, June) and was trailed by *C. japonica* and *P. acrostichoides*; mesic *A. capillus-veneris* had the lowest water content of all four species. Overall, water content in fern leaf tissue did not fluctuate substantially throughout the day.

Average water residence time (*τ_w_)—a function of leaf water content and transpiration rate that shows the amount of time water stays in the bulk leaf water pool (Equation 1)—varied across these species in both May and June (Fig. 5D and 5H, respectively). On both dates, xeric *A. sinuata* not only had the fastest water residence time but it also remained fairly constant at around 1–2 h across diurnal measurements. The other more mesic species had slow water residence times at around 5–10 h, especially in the morning. Water residence time for all species decreased towards midday and midafternoon (Fig. 5D and H).

### Relationship between stomatal response and average water residence time

**Figure 6.**
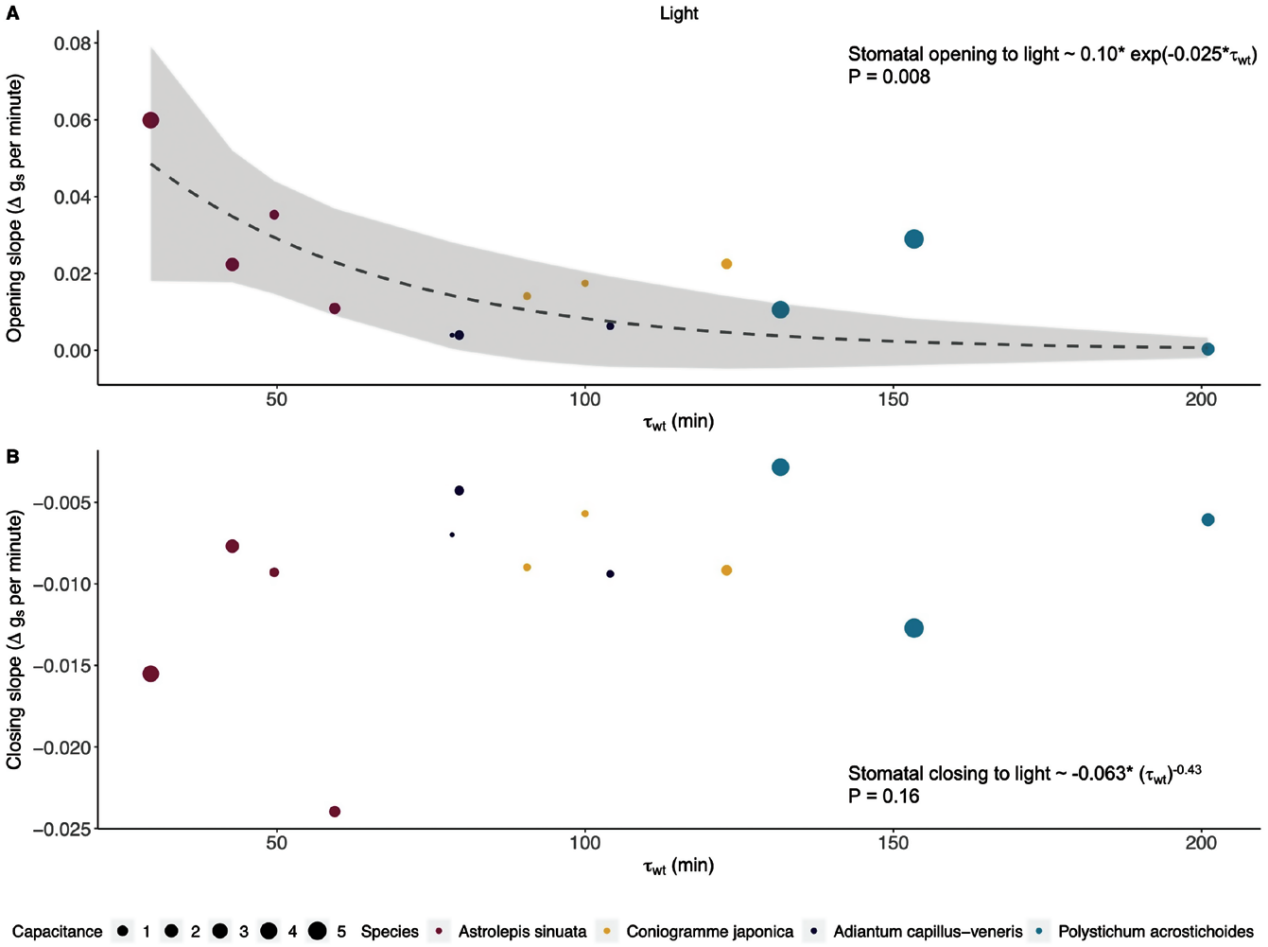
Relationship between average water residence time (*τ*_wt_, in minutes—June data) and stomatal response slopes, with nonlinear least squares regression (grey dashed lines) and 95% confidence intervals (grey shading; Delta method). (A) Stomatal opening to increase in light intensity was significantly related to average water residence time. (B) Stomatal closing to decrease in light intensity was not significantly related to average water residence time. The size of data points reflects the absolute capacitance (*C*_FT_*) of leaves in units of mol m^−2^ MPa^−1^.

Xeric *A. sinuata* had faster stomata responses and average water residence time than the mesic species. We found nonlinear relationships between stomatal response times and average water residence time (Fig. 6). Shorter average water residence time was significantly related to faster stomatal opening in response to light (*P* = 0.008; Fig. 6A; see Supporting Information—Table S6), but not significantly related to stomatal closing rate (*P* = 0.16; Fig. 6B; see Supporting Information—Table S6). Relationships with VPD response are displayed in the supplement ( see Supporting Information—Fig. S9).

## Discussion

While angiosperm stomatal dynamics and water relations are better characterized, fewer studies have examined these relationships in ferns and lycophytes and even fewer have sampled broadly across habits and habitats (i.e. xeric, mesic, epiphytic, terrestrial). Despite the difficulty in assigning taxa to specific habitat characterizations, such as xeric and mesic, especially given the wide geographic distribution for many of them, our vein and stomatal data correlate well with these broad environmental categories. We found anatomical differences related to habits (epiphytic and terrestrial) within ferns and lycophytes and both anatomical and physiological differences across habitats (xeric and mesic) that may help explain environmental preference. Overall, faster stomatal opening responses to light correlated with faster leaf water residence time, suggesting rapidly responding stomata are beneficial for coordinating higher rates of water cycling through leaves.

### Stomatal and vein anatomy

As expected based on previous studies (Carins Murphy *et al.* 2017; Simonin and Roddy 2018), fern and lycophyte vein density was low compared to previously reported values of angiosperms. Indeed, ferns and lycophytes exhibited relatively high stomatal densities given their vein densities (Fig. 1A). Epiphytic ferns and lycophytes had lower vein densities (Fig. 1F) and larger stomata (Fig. 1H) at lower densities (Fig. 1G) than terrestrial species (Fig. 1F, Table 3), but this difference could partially be because these epiphytic species were native to tropical, mesic environments, while our terrestrial species were from more xeric sites. Yet, when the lycophytes were removed and the fern anatomy was analysed in a phylogenetic context, there were no differences between terrestrial and epiphytic species, suggesting that these relationships held across lycophytes but not ferns.

Overall, we found stomatal density did not vary substantially between xeric and mesic studied taxa (Fig. 1D), but vein density was higher in xeric than mesic species (Fig. 1B). While we do not currently understand the developmental and genetic control over these two traits and whether or not they are fundamentally linked, xeric environments may favour higher vein density in xeric ferns. Stomatal density, however, appears to have been unaffected, but stomatal size has been reduced across xeric and mesic ferns and lycophytes (Fig. 1E). Thus, genetic determinants of stomatal density may be more rigidly constrained, while modifications to other parts of the hydraulic pathway may be more labile, allowing these taxa to adapt to the specific environmental constants of xeric habitats. For example, higher vein density in xeric ferns may be more capable of preventing declines in water potential by replenishing water faster than in mesic ferns. Given that mesic ferns live in habitats where they have more frequent access to water, they may not need to have high vein density to meet evaporative demand. However, since our study was not designed to be a true common garden experiment, we cannot fully distinguish the effects of adaptation and plasticity with stomatal or vein traits. Nonetheless, our data show ferns and lycophytes span an impressive range of stomatal densities and xeric ferns had higher vein densities and smaller stomata than mesic ferns, traits that likely allow xeric ferns to supply leaf tissue with enough water to meet evaporative demand in drier environments.

### Stomatal size, response times, and water relations

We expected stomatal size—particularly pore length—to correlate with stomatal response times (Drake *et al.* 2013; Kübarsepp *et al.* 2020); however, within the subset of four Pteridaceae species and one Dryopteridaceae species, stomatal size did not vary greatly, suggesting that stomatal size in the Pteridaceae might be fairly conserved. Yet despite possessing similar stomatal sizes, xeric species did have larger changes in stomatal conductance during step transitions in response to light than mesic species (Fig. 2), which corresponded to faster stomatal opening (Fig. 3). However, there was no difference between xeric and mesic ferns with regard to their stomatal closing responses. Previous studies have shown fern stomata do not close efficiently (McAdam and Brodribb 2012; Elliott-Kingston *et al.* 2016) and even in darkness ferns have substantial stomatal conductance (Creese *et al.* 2014), and our data support those findings for both mesic and xeric species. Mesic ferns may be able to tolerate having slow stomatal responses as they occupy habitats with more consistent access to water, where tight control of water loss may not be as critical as it is for species living in xeric, high-light habitats. Yet, xeric ferns may rely on other strategies besides rapid stomatal closure to survive in dry environments, such as their high vein densities (Fig. 1B) and a suite of leaf anatomical adaptations to help prevent water loss. Desert ferns from the Cheilanthoideae often have leaves with protective scales, hairs, and farina that prevent excessive water loss from leaf tissue, or small pinnae for efficient transpirational cooling (Pickett 1931; Tyron and Tyron 1982; Kirkpatrick 2007; Schuettpelz *et al.* 2007; Hietz 2010); both xeric species from the stomatal response experiments possess a combination of these traits. Additionally, some ferns tolerate dry conditions through drought avoidance by leaf shedding (Hietz 2010), or by being desiccation tolerant and resurrecting when water availability improves, a trait which is found in both epiphytic (Prats and Brodersen 2021) and xeric desert ferns (Holmlund *et al.* 2019). Our data support the idea that xeric ferns would need to rely on a combination of xeric traits rather than rapid stomatal closure (McAdam and Brodribb 2013). While our physiological measurements were only conducted on xeric and mesic species, future studies should incorporate epiphytic and terrestrial ferns and lycophytes into measurements of stomatal dynamics.

In terms of water relations as determined by PV curves, there were no differences between the xeric and mesic species studied, perhaps further suggesting a reliance on other traits for xeric tolerance. Considering stomatal behaviour together with and water residence times, xeric *A. sinuata* had one of the fastest opening and closing times in response to light (Fig. 4) as well as the highest stomatal conductance, the highest water content, and a fast water residence time (Fig. 5). This species also drove the nonlinear relationships between stomatal responses and water residence time (Fig. 6); future work on this topic should include additional xeric species to determine whether this relationship will hold. Yet, relationships between stomatal responses and water residence time suggest an important link between internal leaf anatomy and stomatal closure rates (Fig. 6). Rapid stomatal responses may drive a fast water residence time throughout leaves by exposing the wet internal leaf surfaces to the dry atmosphere. Thus, if stomata open quickly, water begins to evaporate from the pore opening. Conversely, slow stomatal responses allow water to move more slowly through leaves. Stomatal responses and water residence time in the leaf tissue should be studied in more xeric and mesic ferns to finetune these relationships.

## Conclusion

Here, we provide evidence for anatomical and physiological differences in stomatal functioning and water relations between ferns across habitats and habits that together help explain environmental preference. Our study expands the number of ferns and lycophytes for which stomatal anatomy, density, and vein density have been measured. Across ferns and lycophytes there were few veins per stomata, and epiphytic ferns and lycophytes had lower vein densities and larger stomata at lower densities than terrestrial species. Across habitats, xeric ferns had higher vein densities, smaller stomata, and faster stomatal opening responses than mesic species. Physiologically, xeric and mesic species exhibited similar stomatal closing responses to light, aligning with the idea that without hormonally-induced stomatal closing, xeric species need to rely on a suite of other adaptations—including their high vein densities—to survive in dry environments. Furthermore, faster stomatal responses correlated with faster average leaf water residence time, showing the tight link between stomatal response and leaf water relations in these ferns. Altogether, our data show there are anatomical and physiological differences between our studied ferns and lycophytes across habits and habitats, which at least in part likely contribute to environmental preference in their acquisition and utilization of light and water.

## Supporting Information

The following additional information is available in the online version of this article –

**Figure S1**. Cladogram of sampled fern and lycophytes species.

**Figure S2**. Individual light response curves for the subset of five species.

**Figure S3**. Correlation of stomatal width and stomatal length.

**Figure S4**. Average stomatal responses to step changes in VPD across the subset of five species.

**Figure S5**. Stomatal responses to VPD, represented by the slope of the response (change in g_s_ per change in minute).

**Figure S6**. Pressure-volume (PV) curves by species.

**Figure S7**. PV curves for all measured individuals.

**Figure S8**. Water potentials over time during the PV curve dry down.

**Figure S9**. Stomatal response to VPD correlated with average leaf water residence time.

**Table S1.** Parameters from light response curves for each species.

**Table S2**. Linear regressions between vein density and stomatal density for mesic and xeric ferns and lycophytes.

**Table S3**. Summary output from phylogenetic ANOVAs on just the fern anatomy dataset.

**Table S4**. Summary output from phylogenetic ANOVAs conduced on species means from the subset of five species.

**Table S5**. Linear regression results for stomatal anatomy and stomatal responses to light intensity and VPD.

**Table S6**. Non-linear relationships between water turnover and stomatal response slopes.

plae041_suppl_Supplementary_Figures_S1-S9_Tables_S1-S6

plae041_suppl_Supplementary_Data

## Data Availability

All data (species means) have been provided in the Supplemental Spreadsheet “Species Means”.
