# Supplementary material for: Stomatal behaviour and water relations in ferns and lycophytes across habits and habitats
Source: AoB Plants. 2024 Jul 20;16(4):plae041. doi: 10.1093/aobpla/plae041 (PMC11306579; doi:10.1093/aobpla/plae041)
Supplement: plae041_suppl_Supplementary_Figures_S1-S9_Tables_S1-S6 [file plae041_suppl_supplementary_figures_s1-s9_tables_s1-s6.docx]

**Supplementary Materials for article “*Stomatal behavior and water relations in ferns and lycophytes across habits and habitats”***

**Methods**

*Stomatal response time to VPD (applies to the VPD responses displayed in Figures S4, S5, and S9 below)*

We measured stomatal responses to VPD on three to four individuals of *Adiantum capillus-veneris*, *Astrolepis sinuata*, *Coniogramme japonica*, and *Cheilanthes distans* using a LI-6800 (Li-Cor, Lincoln, NE, USA). The LI-6800 was set to maintain 415 µmol mol^-1^ CO_2_ in the sample chamber, leaf temperature at 25°C, 600 µmol s^-1^ flow, and 250 µmol (photons) m^-2^ s^-1^ in the chamber head. VPD was initially set to 1.5 kPa for 10 minutes to allow stomatal conductance to stabilize, then it was increased to 2.5 kPa for 30 minutes to measure stomatal closing rates. Finally, VPD was reduced back to 1.5 kPa for 30 minutes to measure stomatal opening rates. To account for the time lags in flow and mixing of the gas exchange system after step changes in VPD, we removed the first five minutes of data after each step change in VPD. Similar to the light response, leaf areas of fern pinnae in the chamber were measured in ImageJ to recalculate gas exchange parameters. Rates of stomatal opening and closing in response to VPD step changes were calculated by first normalizing *g_s_* to the maximum value for each species, and then calculating the slope of stomatal response from the initial step change (again excluding 5 minutes after LI-6800 flow adjustments) to the point at which the *g_s_* leveled off and the rate of change between measurements was ≤ 2%. We used at least 10 measurements for each VPD opening and closing slope calculation.

**Figures**


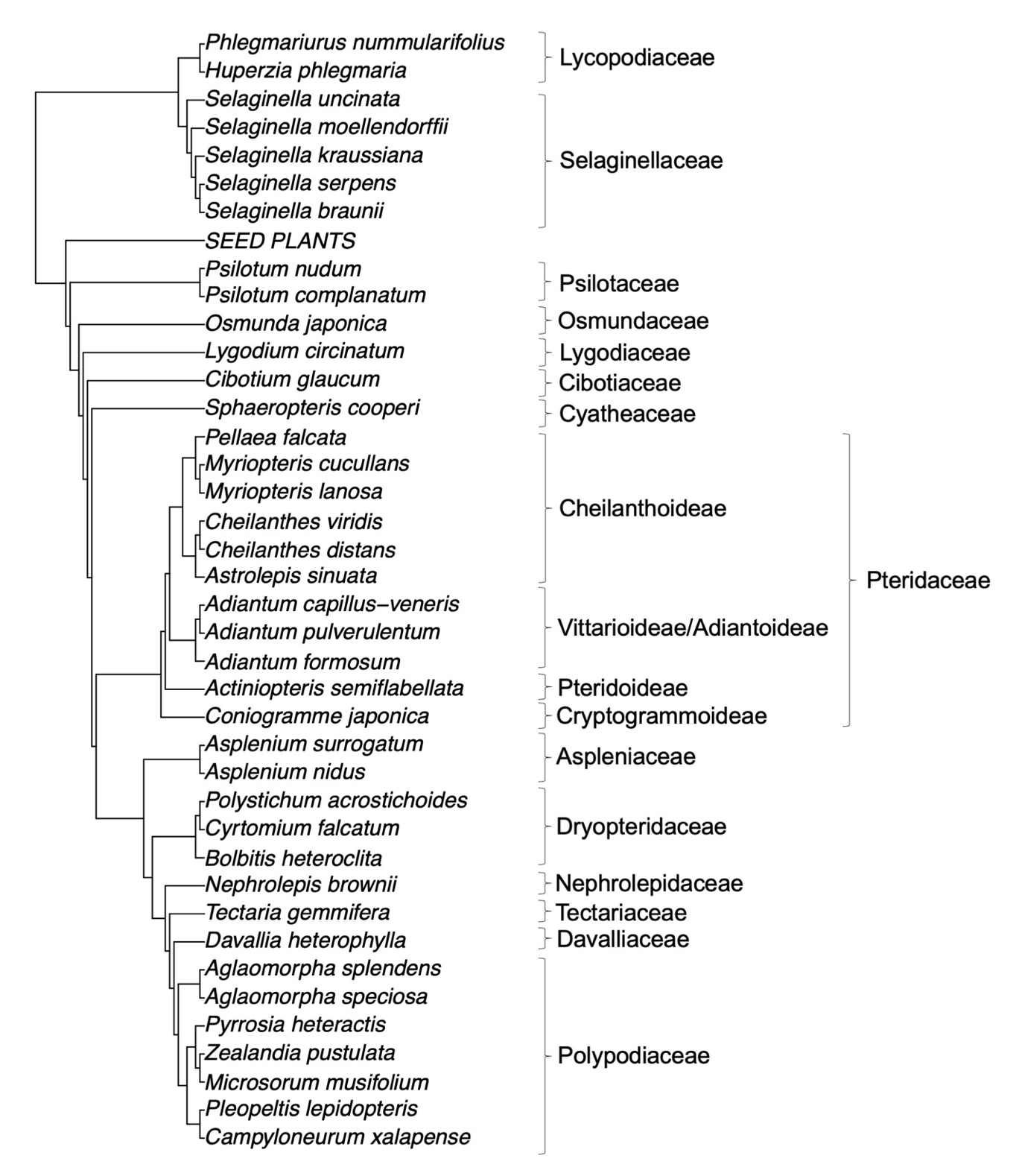


**Figure S1**. Cladogram of sampled fern and lycophytes species, with corresponding family and/or subfamily information. Seed plants are shown as sister to the ferns. The cladogram was constructed by consulting with established fern and lycophyte phylogenies (Schuettpelz et al., 2007; Schuettpelz and Pryer, 2007; Schuettpelz et al., 2016) and using R packages ‘ape’ (Paradis and Schliep, 2019), ‘phangorn’ (Schliep et al., 2017), ‘phytools’ (Revell, 2012), and ‘geiger’ (Pennell et al., 2014).


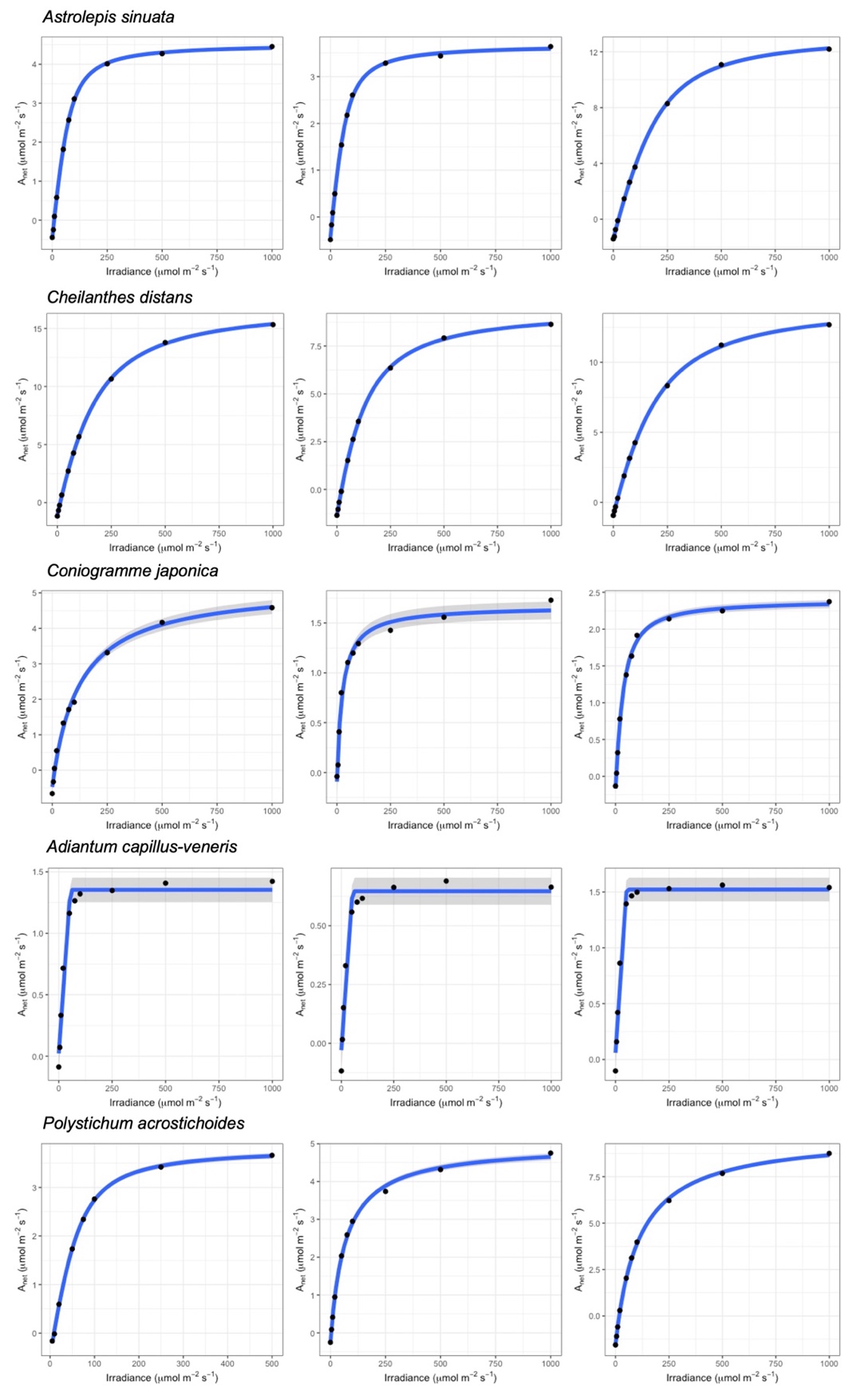


**Figure S2**. Individual light response curves for each species. Nonlinear regression models (derived from (Marshall and Biscoe, 1980)) were fit using the ‘photosynthesis’ package (Stinziano et al., 2021). The y-axes differ in their limits.

**Figure S3**. Stomatal width correlated with stomatal length (n = 39, Linear regression: R^2^ = 0.48, F(1, 37) = 35.7, P < 0.00001). Fern and lycophyte values from the literature (green open diamonds) were excluded from these statistical analyses. Labels are included for the five species from stomatal response and water relations measurements (As = *Astrolepis sinuata*, Cd = *Cheilanthes distans*, Cj = *Coniogramme japonica*, Ac-v = *Adiantum capillus-veneris*, and Pa = *Polystichum acrostichoides*).The dashed gray line shows the regression fit, while the light gray shaded region is the 95% confidence interval.


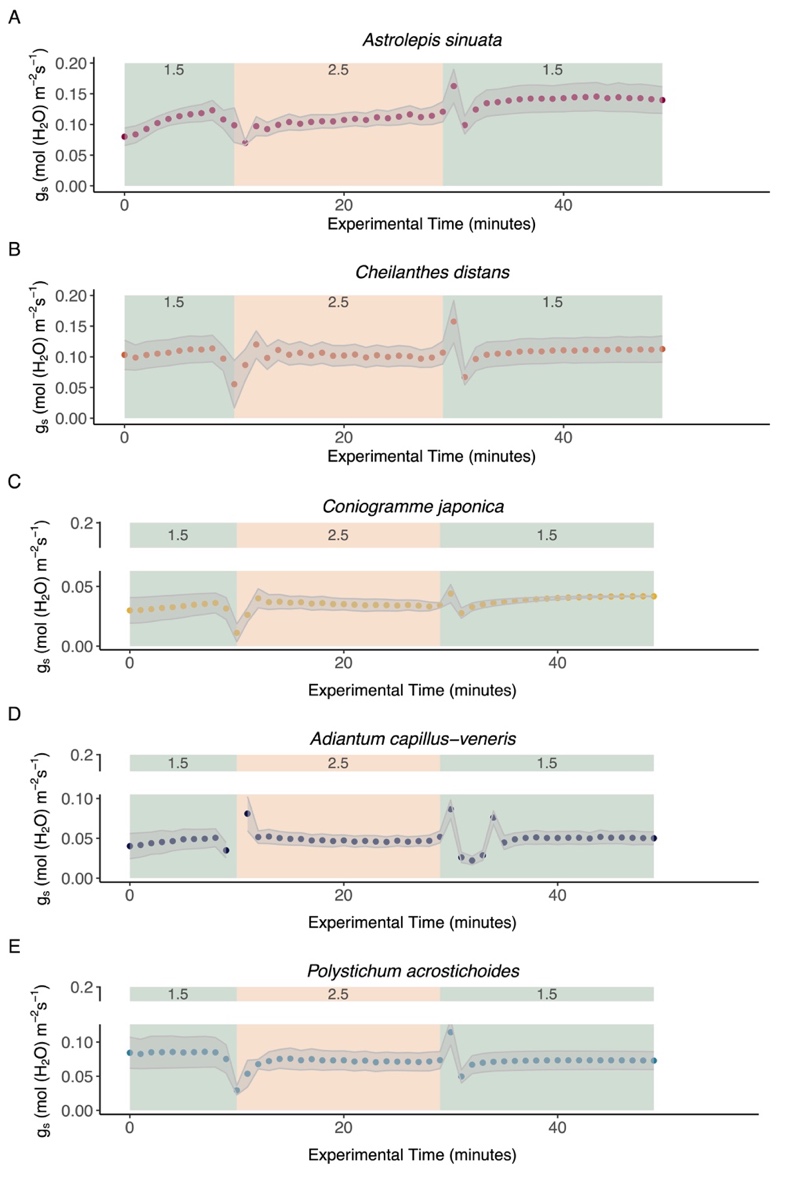


**Figure S4**. Average stomatal responses to step changes in VPD across five species (xeric *Astrolepis sinuata* (n = 4), xeric *Cheilanthes distans* (n = 3), mesic *Coniogramme japonica* (n = 3), mesic *Adiantum capillus-veneris* (n = 3), and mesic *Polystichum acrostichoides* (n = 3)). Gray shading represents standard error around the mean (colored points). Background colors show the low (green) and high (orange) VPD conditions, with corresponding numbers displaying the VPD values. Axis breaks (Xu *et al.* 2021) are shown to provide more detail for the mesic species that had lower fluxes compared to the xeric species.


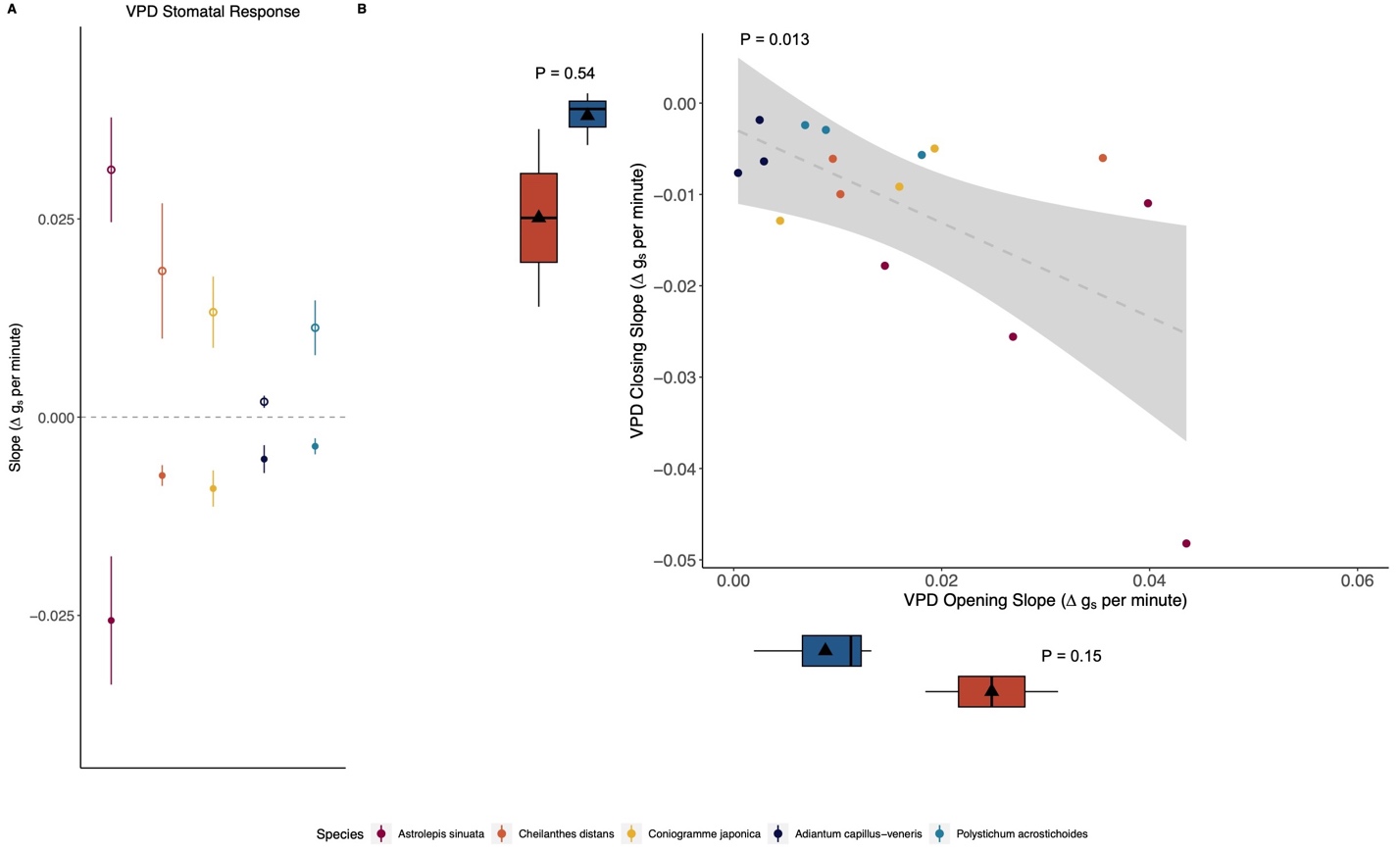


**Figure S5**. A) Stomatal opening (open circles) and closing (closed circles) response to VPD for the five species (xeric *Astrolepis sinuata* (n = 4), xeric *Cheilanthes distans* (n = 3), mesic *Coniogramme japonica* (n = 3), mesic *Adiantum capillus-veneris* (n = 3), and mesic *Polystichum acrostichoides* (n = 3)). Stomatal responses are represented by the slope of the response (change in g_s_ per change in minute). Positive slopes signify stomatal opening (open circles), and negative slopes signify stomatal closing (closed circles); the higher the absolute value, the larger the slope, and thus, the quicker the stomatal response. B) Opening and closing rates to VPD stimuli were significantly correlated (P = 0.013), yet there were no differences between xeric and mesic ferns with stomatal opening (boxplot along x-axis) or closing (boxplot along y-axis) in response to VPD (P-values from phylogenetic ANOVAs). In all xeric (n = 7) and mesic (n = 9) boxplots, the triangles represent the mean, the center lines are the median, the box limits are the upper and lower quartiles, the whiskers are 1.5x interquartile range.

**Figure S6**. Pressure-volume (PV) curves by species (xeric *Astrolepis sinuata* (n = 4), xeric *Cheilanthes distans* (n = 4), mesic *Coniogramme japonica* (n = 4), mesic *Adiantum capillus-veneris* (n = 4), and mesic *Polystichum acrostichoides* (n = 4)). Symbols represent different fronds during the PV curve dry downs. The y-axes have differing limits.


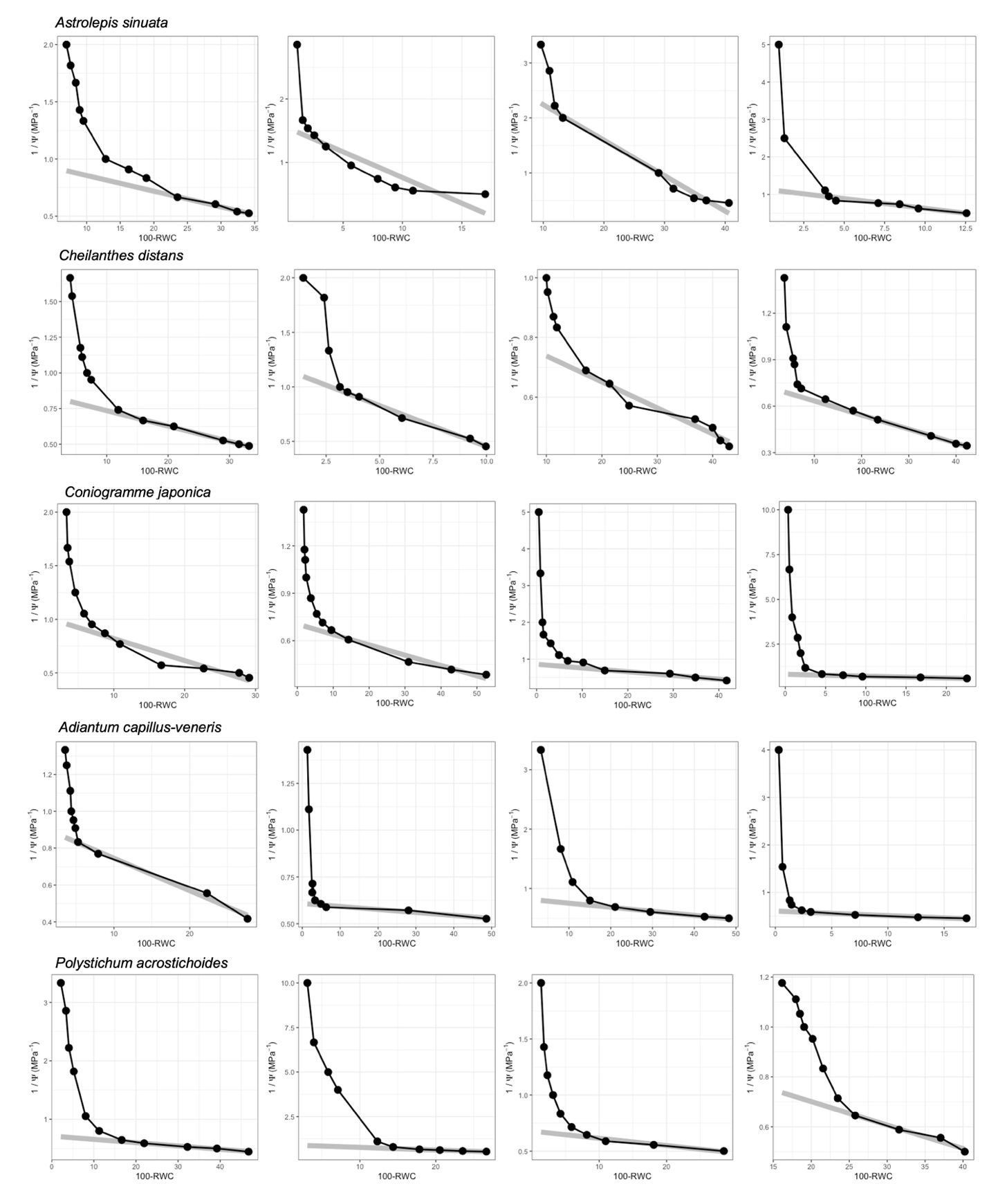


**Figure S7.** PV curves for all measured individuals. Gray lines show the extrapolated linear relationship between 1/Ψ and 100-RWC after the turgor loss point. The y-axes are plotted with different limits. Models were fit using the ‘photosynthesis’ package to determine parameters (Stinziano et al., 2021).

**Figure S8.** Water potentials over time during the PV curve dry down (xeric *Astrolepis sinuata* (n = 4), xeric *Cheilanthes distans* (n = 4), mesic *Coniogramme japonica* (n = 4), mesic *Adiantum capillus-veneris* (n = 4), and mesic *Polystichum acrostichoides* (n = 4))Symbols represent different fronds during the PV curve dry downs.


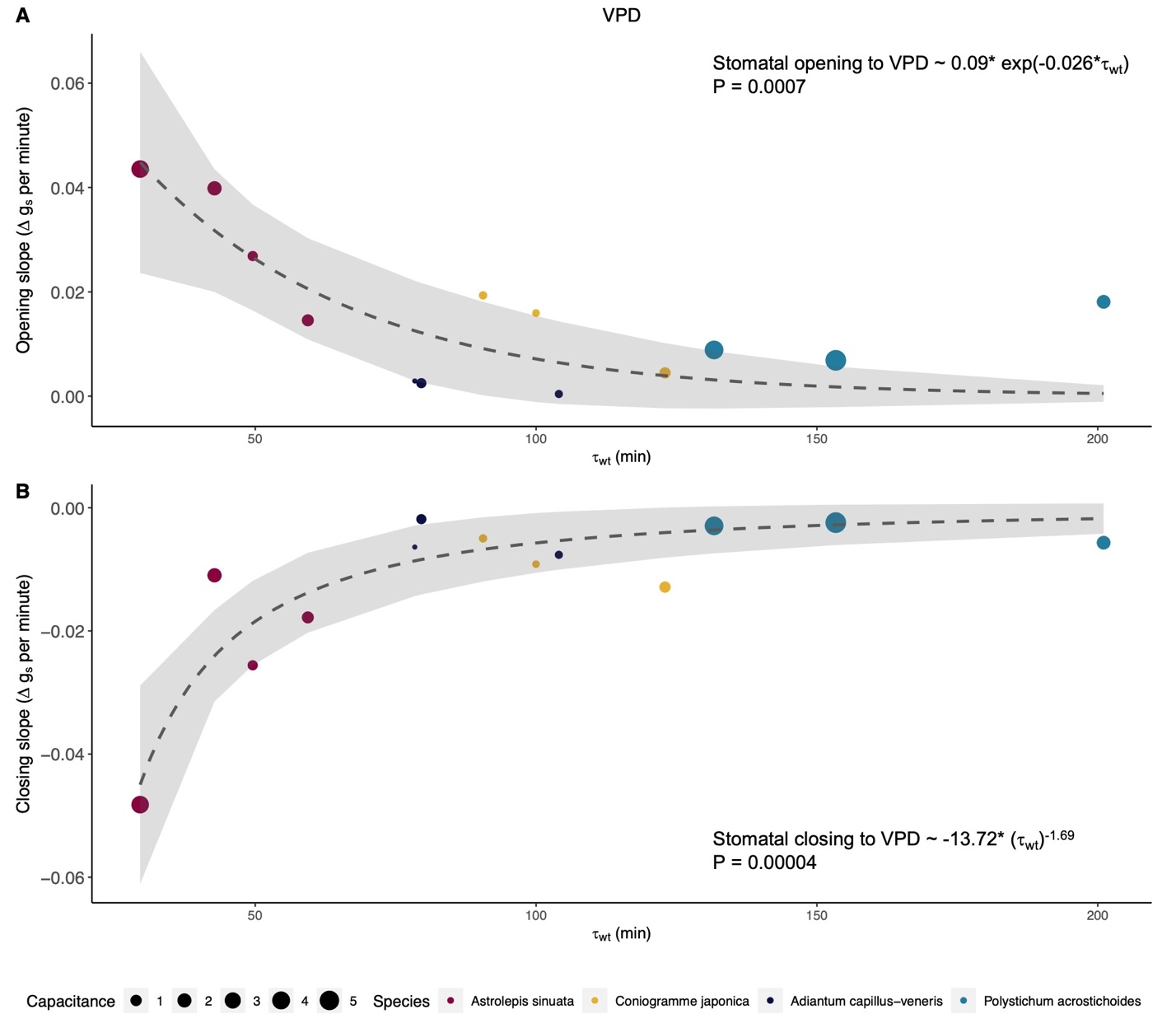


**Figure S9**. Both stomatal opening to VPD decrease (A) and stomatal closing to VPD increase (B) were significantly related to average water residence time. The size of data points reflects the absolute capacitance (C_FT_*) of leaves in units of mol m^-2^ MPa^-1^.

**Tables**

**Table S1.** Parameters from light response curves for each species. Nonlinear regression models (derived from (Marshall and Biscoe, 1980)) were fit using the ‘photosynthesis’ package (Stinziano et al., 2021). *A_sat_* is the photosynthetic rate in µmol CO_2_ m^-2^ s^-1^ at light saturation, Φ_J_ is the quantum yield of CO_2_ assimilation, θ_J_ is the curvature of the light response curve, *R_D_* is the dark respiration rate in µmol CO_2_ m^-2^ s^-1^, *I_comp_* is the light compensation point in µmol photons m^-2^ s^-1^, and RSS is the residual sum of squares.

| **Species** | ***A_sat_*** | | **Φ_J_** | | **θ_J_** | | ***R_D_*** | | ***I_comp_*** | | **RSS** | |
| --- | --- | --- | --- | --- | --- | --- | --- | --- | --- | --- | --- | --- |
| *Astrolepis sinuata* | 7.95 ± 5.89 | | 0.05 ± 0.006 | | 0.75 ± 0.02 | | 0.77 ± 0.54 | | 13.93 ± 8.80 | | 0.03 ± 0.04 | |
|  |  | 4.99 |  | 0.05 |  | 0.77 |  | 0.47 |  | 8.69 |  | 0.008 |
|  |  | 4.12 |  | 0.05 |  | 0.75 |  | 0.44 |  | 9.00 |  | 0.01 |
|  |  | 14.73 |  | 0.06 |  | 0.73 |  | 1.39 |  | 24.09 |  | 0.07 |
| *Cheilanthes distans* | 14.75 ± 3.72 | | 0.07 ± 0.01 | | 0.56 ± 0.02 | | 1.12 ± 0.22 | | 16.60 ± 4.33 | | 0.03 ± 0.02 | |
|  |  | 10.8 |  | 0.07 |  | 0.54 |  | 1.35 |  | 21.45 |  | 0.01 |
|  |  | 15.27 |  | 0.06 |  | 0.58 |  | 0.92 |  | 15.21 |  | 0.03 |
|  |  | 18.18 |  | 0.08 |  | 0.57 |  | 1.08 |  | 13.13 |  | 0.04 |
| *Coniogramme japonica* | 3.33 ± 2.07 | | 0.06 ± 0.01 | | 0.18 ± 0.28 | | 0.24 ± 0.20 | | 5.12 ± 5.05 | | 0.07 ± 0.07 | |
|  |  | 5.67 |  | 0.05 |  | < 0.001 |  | 0.47 |  | 10.86 |  | 0.15 |
|  |  | 1.76 |  | 0.07 |  | 0.03 |  | 0.09 |  | 1.39 |  | 0.05 |
|  |  | 2.56 |  | 0.06 |  | 0.50 |  | 0.17 |  | 3.10 |  | 0.02 |
| *Adiantum capillus-veneris* | 1.18 ± 0.44 | | 0.02 ± 0.01 | | 1 ± 0 | | 0.01 ± 0.02 | | 0.76 ± 1.31 | | 0.08 ± 0.05 | |
|  |  | 0.68 |  | 0.01 |  | 1 |  | 0.03 |  | 2.27 |  | 0.03 |
|  |  | 1.35 |  | 0.02 |  | 1 |  | 0 |  | 0 |  | 0.09 |
|  |  | 1.50 |  | 0.03 |  | 1 |  | 0 |  | 0 |  | 0.12 |
| *Polystichum acrostichoides* | 6.94 ± 3.79 | | 0.08 ± 0.02 | | 0.25 ± 0.43 | | 0.76 ± 0.68 | | 9.78 ± 6.85 | | 0.03 ± 0.02 | |
|  |  | 5.23 |  | 0.08 |  | 0.0009 |  | 0.26 |  | 3.44 |  | 0.04 |
|  |  | 11.29 |  | 0.10 |  | 0.0002 |  | 1.53 |  | 17.04 |  | 0.05 |
|  |  | 4.31 |  | 0.06 |  | 0.75 |  | 0.48 |  | 8.86 |  | 0.01 |

**Table S2**. Linear regressions between vein density and stomatal density for mesic and xeric ferns and lycophytes. Mesic regression line is plotted in Figure 1.

| **Response variable)** | **Explanatory variable)** | **Adjusted R^2^** | **F** | **T** | **P** | **RSE** |
| --- | --- | --- | --- | --- | --- | --- |
| Mesic species vein density | Mesic species stomatal density (log-transformed) | 0.49 | F(1, 24) = 24.7 |  | P < 0.0001*** |  |
| Xeric species vein density | Xeric species stomatal density (log-transformed) | -0.19 | F(1, 5) = 0.04 |  | 0.85 |  |

**Table** **S3**. Summary output from phylogenetic ANOVAs on just the fern anatomy dataset (stomatal density, vein density, and stomatal size) for habitat (xeric, mesic) and habit (terrestrial, epiphytic).

|  | **Trait** | **F (1,25)** | **Z** | **P-value** |
| --- | --- | --- | --- | --- |
| Habitat (xeric vs. mesic) | Stomatal density (mm^-2^) | 0.001 | -2.04 | 0.97 |
|  | Stomatal size (µm^2^) | 8.856 | 2.34 | 0.01 * |
|  | Vein density (mm mm^-2^) | 4.028 | 1.61 | 0.05 * |
| Habit (terrestrial vs. epiphytic) | Stomatal density (mm^-2^) | 0.053 | -0.88 | 0.82 |
|  | Stomatal size (µm^2^) | 0.969 | 0.48 | 0.33 |
|  | Vein density (mm mm^-2^) | 0.431 | -0.02 | 0.52 |

**Table S4**. Summary output from phylogenetic ANOVAs conduced on species means from the subset of xeric ferns (*Astrolepis sinuata, Cheilanthes distans*; n = 7 individuals) vs. mesic ferns (*Coniogramme japonica, Polystichum acrostichoides, Adiantum capillus-veneris*; n = 9 individuals). Data are plotted in Figures 3 and 4.

| **Variables** | **Traits** | **F (1,4)** | **Z** | **P-value** |
| --- | --- | --- | --- | --- |
| Xeric vs. mesic stomatal response slopes | Light opening slope | 11.74 | 1.69 | 0.05 * |
|  | Light closing slope | 0.51 | -0.10 | 0.59 |
|  | VPD opening slope | 2.81 | 0.94 | 0.15 |
|  | VPD closing slope | 0.67 | 0.05 | 0.54 |
| Xeric vs. mesic water relations (from PV curves) | TLP | 1.84 | 0.59 | 0.31 |
|  | SWC | 1.14 | 0.26 | 0.44 |
|  | RWC_TLP_ | 3.41 | 0.88 | 0.15 |
|  | C_FT_* | 0.17 | -0.32 | 0.63 |

**Table S5**. Linear regression results for stomatal anatomy and stomatal responses to light intensity and VPD. The RSE is the residual standard error.

| **Stomatal response (response variable)** | **Anatomy (explanatory variable)** | **Adjusted R^2^** | **F** | **T** | **P** | **RSE** |
| --- | --- | --- | --- | --- | --- | --- |
| Light opening slope | Stomatal pore length | -0.31 | F(1, 3) = 0.06 | -0.25 | 0.82 | 0.02 |
|  | Guard cell length | -0.33 | F(1, 3) < 0.001 | 0.84 | 0.98 | 0.01 |
|  | Stomatal density | -0.29 | F(1, 3) = 0.08 | 0.29 | 0.79 | 0.02 |
| Light closing slope | Stomatal pore length | -0.31 | F(1, 3) = 0.05 | 0.22 | 0.84 | 0.003 |
|  | Guard cell length | -0.33 | F(1, 3) = 0.002 | 0.05 | 0.96 | 0.005 |
|  | Stomatal density | 0.45 | F(1, 3) = 4.3 | 2.07 | 0.13 | 0.002 |
| VPD opening slope | Stomatal pore length | -0.32 | F(1, 3) = 0.02 | -0.14 | 0.89 | 0.01 |
|  | Guard cell length | -0.33 | F(1, 3) = 0.01 | 0.13 | 0.91 | 0.01 |
|  | Stomatal density | -0.15 | F(1, 3) = 0.48 | -0.69 | 0.54 | 0.01 |
| VPD closing slope | Stomatal pore length | -0.32 | F(1, 3) = 0.02 | 0.15 | 0.89 | 0.01 |
|  | Guard cell length | -0.33 | F(1, 3) < 0.001 | 0.009 | 0.99 | 0.01 |
|  | Stomatal density | 0.38 | F(1, 3) = 3.49 | 1.87 | 0.16 | 0.006 |

**Table S6**. Non-linear relationships between water turnover and stomatal response slopes. Data are plotted in Figure 6.

| **Variables** | **Equation** | **Residual Sum of Squares (RSS)** | **Pearson’s product-moment correlation** | **T** | **DF** | **P-value** |
| --- | --- | --- | --- | --- | --- | --- |
| Water turnover time (τ_wt_) vs. stomatal response slopes | Stomatal opening to light ~ 0.10* exp(-0.025*τ_wt_) | 0.0019 | 0.70 | 3.22 | 11 | 0.008 ** |
|  | Stomatal closing to light ~ -0.063* (τ_wt_)^-0.43^ | 0.0003 | 0.41 | 1.50 | 11 | 0.16 |
|  | Stomatal opening to VPD ~ 0.09* exp(-0.026*τ_wt_) | 0.0008 | 0.81 | 4.66 | 11 | 0.0007*** |
|  | Stomatal closing to VPD ~ -13.72* (τ_wt_)^-1.69^ | 0.0004 | 0.89 | 6.57 | 11 | 0.00004 *** |
